# Bibliometric analysis of worldwide publications on multi-, extensively, and totally drug – resistant tuberculosis (2006–2015)

**DOI:** 10.1186/s40248-016-0081-0

**Published:** 2017-01-11

**Authors:** Waleed M. Sweileh, Adham S. AbuTaha, Ansam F. Sawalha, Suleiman Al-Khalil, Samah W. Al-Jabi, Sa’ed H. Zyoud

**Affiliations:** 1 Department of Pharmacology/ Toxicology, College of Medicine and Health Sciences, An-Najah National University, Nablus, 44839 Palestine; 2 Department of Anatomy, Biochemistry and Genetics, College of Medicine and Health Sciences, An-Najah National University, Nablus, 44839 Palestine; 3 Department of Clinical and Community Pharmacy, College of Medicine and Health Sciences, An-Najah National University, Nablus, 44839 Palestine

**Keywords:** Tuberculosis, Drug Resistance, Bibliometrics

## Abstract

**Background:**

The year 2015 marked the end of United Nations Millennium Development Goals which was aimed at halting and reversing worldwide tuberculosis (TB). The emergence of drug resistance is a major challenge for worldwide TB control. The aim of this study was to give a bibliometric overview of publications on multi-, extensively, and totally drug-resistant TB.

**Methods:**

Scopus database was used to retrieve articles on multidrug resistant (MDR), extensively drug-resistant (XDR), and totally drug-resistant (TDR) tuberculosis for the study period (2006–2015). The number of publications, top productive countries and institutions, citation analysis, co-authorships, international collaboration, active authors, and active journals were retrieved and analyzed.

**Results:**

A total of 2260 journal articles were retrieved. The mean ± SD citations per article was 7.04 ± 16.0. The *h*-index of retrieved data was 76. The number of publications showed a three – fold increase over the study period compared with less than two – fold increase in tuberculosis research during the same study period. Stratified by number of publications, the United States of America ranked first while Switzerland ranked first in productivity per 100 million people, and South Africa ranked first in productivity stratified per one trillion Gross Domestic Product. Three of the High Burden Countries (HBC) MDR-TB (India, China, and South Africa) were present in top productive countries. High percentage of international collaboration was seen among most HBC MDR-TB. Except for *Plos One* journal, most active journals in publishing articles on MDR, XDR, TDR-TB were in infection – related fields and in general medicine. Top 20 cited articles were published in prestigious journal such as *Lancet* and *New England Journal of Medicine*. The themes in top 20 cited articles were diverse, ranging from molecular biology, diagnostic tools, co-infection with HIV, and results of new anti-TB drugs.

**Conclusion:**

Publications on MDR, XDR and TDR – TB are increasing in the past decade. International collaboration was common. Many low resourced African and Asian countries will benefit from research leading to new diagnostic and screening technology of TB. The exchange of expertise, ideas and technology is of paramount importance in this field.

## Background

Tuberculosis (TB) is considered a top infectious disease killer worldwide making it a major public health problem [1, 2]. Global Tuberculosis Report, published by World Health Organization (WHO), stated that in 2014, TB killed 1.5 million people [3]. The year 2015 marked the end of United Nations Millennium Development Goals (MDGs) which was aimed at halting and reversing worldwide TB, malaria and human immunodeficiency virus (HIV). Despite the continuous and slow decline in TB, there are still few challenges confronting the control of TB. Association of TB with poverty, co-infection with HIV, and emergence of TB drug resistance are major challenges for worldwide TB control [2]. Antimicrobial resistance (AMR), in general, is a global challenge that threatens the clinical benefit of many important antimicrobial agents and requires an immediate action for containment and reversal [4]. In case of TB, three serious types of drug resistant TB have been identified: (1) multidrug-resistant TB (MDR-TB) which is a TB with resistance to at least isoniazid and rifampicin; (2) extensively drug-resistant TB (XDR-TB), which is a form of MDR-TB that is resistant to isoniazid and rifampin, plus any fluoroquinolone and at least one of three injectable second-line drugs (i.e., amikacin, kanamycin, or capreomycin) [5, 6]; and finally, total drug-resistant TB (TDR) in which *Mycobacterium tuberculosis* strains are resistant to all first and second line anti-TB agents [7–10].

In 2014, MDR-TB was responsible for the death of 190,000 people and it is predicted that if all TB cases identified in 2014 were tested for drug resistance, a total of 300,000 patients would carry MDR-TB. More than half (54%) of these patients are living in India, China and Russian Federation [3]. Treatment and control of TB requires the availability of effective drug therapy which in turn requires understanding the pattern of growth and development of resistance of *Mycobacterium tuberculosis* to first line drug therapy. The WHO has developed the concept of “High Burden Country” (HBC) to emphasize the health burden and to facilitate the understanding of global TB burden post 2015 era. The HBC MDR -TB list includes 20 HBC countries in terms of absolute numbers of cases and an additional 10 countries with the most severe burden in terms of case rates *per capita* that do not already appear in the “top 20” [3, 11].

Research on drug resistance is a key element for future planning to eliminate TB since the progress made on halting TB in the past two decades could be undermined by the development of drug-resistant *Mycobacterium tuberculosis* [12]. This requires evaluation and analysis of published research on resistant TB and where we will stand on this issue in the future. Publications on TB and the contribution of various countries to these publications have been studied through bibliometric studies [13–17]. However, no such studies, that the author is aware of, have described the bibliometrics of drug resistant TB in general and MDR, XDR, and TDR-Tb in specific. In fact, one of the components of *Stop TB Strategy* and *End TB Strategy* is intensified research and innovation [3].

Bibliometrics is a statistical method used to analyze and assess various aspects related to the topic of interest [18]. Such aspects include contribution of countries, core journals, most active researchers, international collaboration, annual growth of publications, and top cited articles in the field [19]. This study was carried out as an effort to shed light on a challenging worldwide problem facing the future target to end TB. The objective of this study was to retrieve and analyze worldwide publications on MDR, XDR and TDR-TB for the past decade (2006 – 2015). In this study, bibliometric indicators for data extracted from Scopus database will be presented and gaps pertaining to knowledge about research activity on MDR, XDR and TDR-TB will be filled.

## Methods

The methodology used in this study has been described in previously published bibliometric studies [20–28]. Several electronic databases can be used to carry out bibliometric studies. Scopus database has been selected to perform this study because it has several advantages over other databases [29]. Scopus includes larger number of journals than either Pubmed or Web of Science. Furthermore, Scopus allows a wide range and more accurate data analysis than either Pubmed or Google scholar [30].

**Fig. 1:**
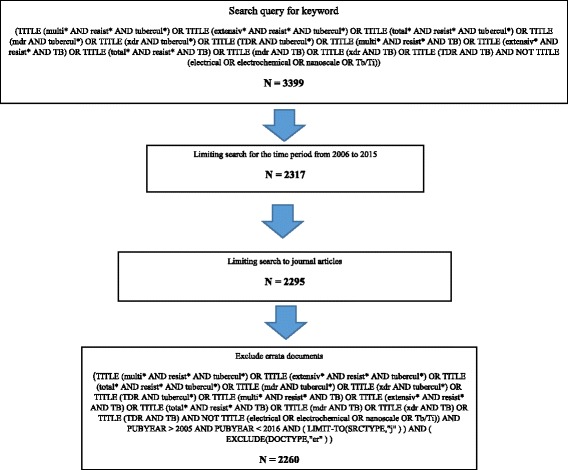
Scheme showing steps of search strategy with keywords used and numbers of articles retrieved in each step

In this study, data were retrieved using keywords in article title to maximize accuracy of the results. The keywords used in this study were obtained partially from previous review articles on MDR-TB [31]. The use of keywords in title-abstract search will yield a high percentage of false positive results. However, the use of keywords in title search only will minimize false positive results. Figure 1 shows a scheme that includes search queries used and the number of retrieved articles in each step.

In search query shown in Fig. 1, the asterisk was used after some keywords as a wild card to retrieve all words relevant to the keyword of interest. The duration of study was set from 2006 to 2015. The study period represents one decade. During this period reports of MDR, XDR, and TDR-TB has emerged strongly. The retrieved documents were limited to journal articles while books and book chapters were excluded. No exclusion was made based on language of the document.

The validity of search strategy was tested by manually reviewing a random sample of 20% of retrieved articles. Validity was confirmed when all randomly tested articles were related to MDR, XDR or TDR-TB. The validation test confirmed that there were minimum false positive results in retrieved documents. Three articles that were false positive were excluded using the function “AND NOT” as appeared in the search query (Fig. 1). However, false negative results remain a possibility especially when the search query was based on title search only. The author, with the help of experts in data management, tried at least five different scenarios of search queries using different search strategies, and the ultimate results in all scenarios had large numbers of false positive results with no significant addition of true articles on MDR, XDR, and TDR-TB. Therefore, the use of the current search query will avoid large numbers of false positive articles but will miss very few true articles.

The growth of research on MDR, XDR, and TDR - TB was presented graphically using Statistical Package for Social Sciences (SPSS). The contribution of countries and institutions/organizations was limited to top ten or those with a minimum productivity of 20 articles. Productivity of HBC TDR-TB was also presented. The standard competition ranking (SCR) was used to rank top ten productive countries, institutions, and authors. Data pertaining to productivity by countries was also stratified with population size and Gross Domestic Product (GDP). For each country, the total number of population and GDP were obtained from World Bank website. To calculate the productivity of each country stratified by population size, we divided the number of publications by the population size of that country. Similarly, to calculate the productivity stratified by GDP, we divided the number of publications by GDP of that country. For population size, productivity was calculated per 100 million inhabitants while for GDP, productivity was calculated per one trillion GDP.

Scopus can be used to provide analysis on international collaboration by counting number of articles published by authors from the same country and articles with authors from different countries. In other words, there are two types of articles: single country publications (SCP) in which all authors belong to the same country and such publications represent intra-country collaboration; and multiple country publications (MCP) in which authors belong to different countries and such publications represent inter-country collaboration i.e. international collaboration.

Quality of publications is difficult to measure or assess directly. However, total number of citations received, average number of citations per article, Hirsch-index (*h*-index), percentage of highly cited articles, and impact factor (IF) of journals can be used as an indirect measure of publication impact or quality. H-index has been developed to assess productivity and citation impact of researchers. However, the use of *h*-index has been extended to measure the productivity and citation impact of countries and academic institutions [32]. In this study, *h*-indexes for countries and institutions were obtained directly from Scopus database while IF was obtained from Journal Citation Report 2015 published by Thomson Reuters. To visualize country collaboration and co-authorships, VOSviewer was used [33]. VOSviewer can present information either as density visualizations or network visualizations maps.

Ethical approval of this study was not required by IRB since no human subjects or data were involved. All data analysis was carried out on November 13^th^, 2016 to avoid the dynamics of citations from 1 day to another.

## Results

### General information

**Fig. 2:**
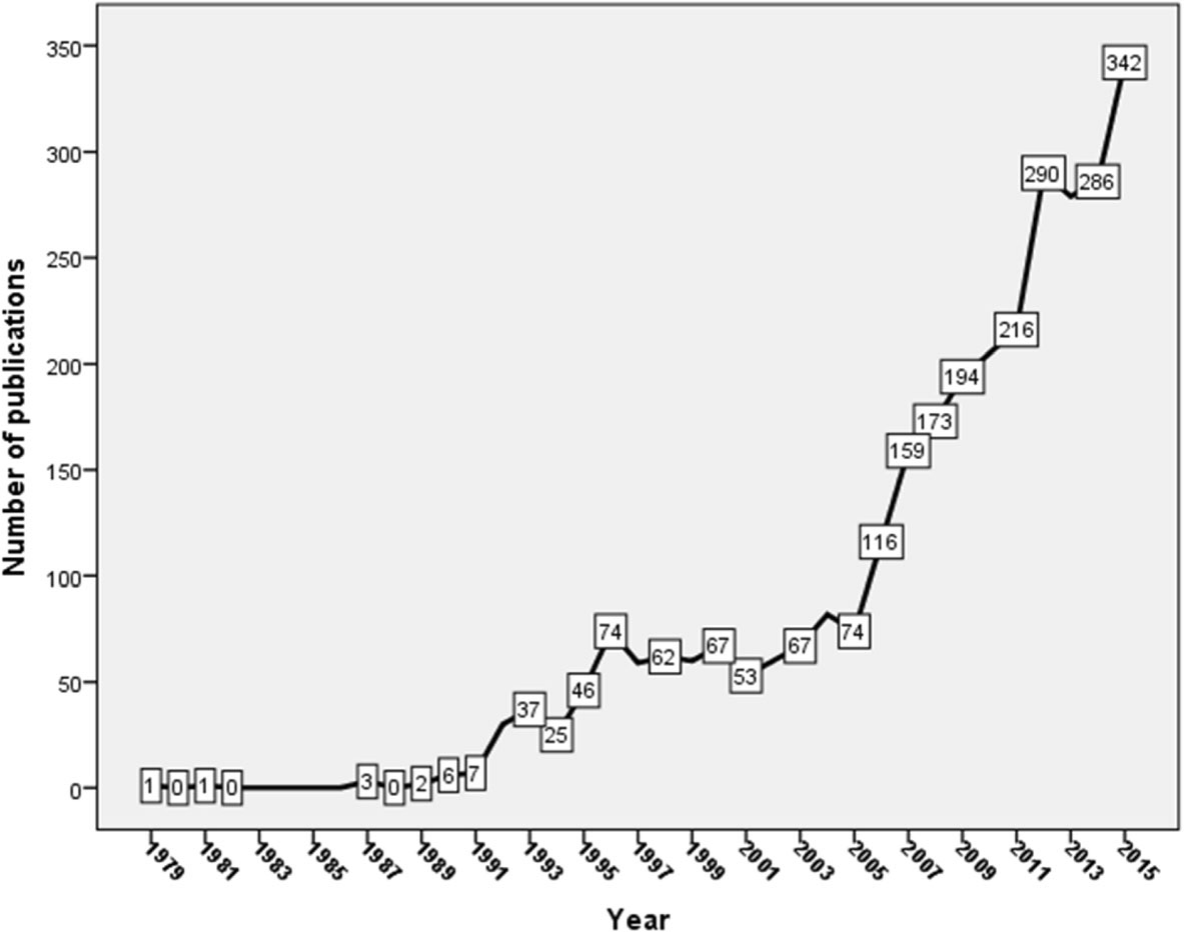
Growth of publications on MDR, XDR and TDR-TB in the past four decades

**Fig. 3:**
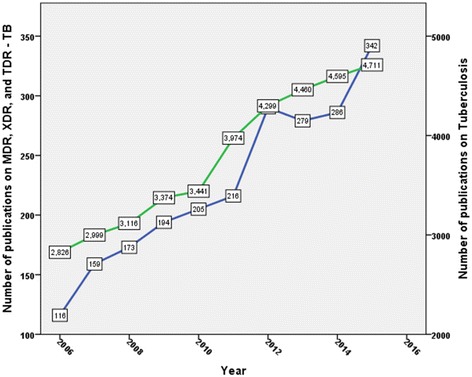
Growth of worldwide publications on TB and on MDR, XDR, TDR-TB during the past decade

Two thousand two hundred sixty journal articles were retrieved. The majority of retrieved documents were research articles (1612; 71.3%). Other types of retrieved documents are shown in Table 1. English (2047; 90.57%) was the most commonly encountered language followed by Spanish (49; 2.17%) and Chinese (45; 2.0%). The total number of citations received was 35,661 (mean ± SD = 7.04 ± 16.0; median (Q1-Q3) = 3(0–7)) while *h*-index was 76. The highest number of publications was recorded in 2015 with a total of 342, while the lowest number of publications was recorded in 2006 with a total number of 116. This represents a threefold increase in number of publications. Figure 2 shows the growth of publications on the past four decades while Fig. 3 shows the growth of publications on tuberculosis in general and on MDR, XDR, and TDR resistance in particular in the past decade. The growth of publications on TB showed less than two- fold increase in the past decade while the growth of publications on MDR, XDR, and TDR – TB showed approximately three – fold increase.

**Table 1:** Types of retreived documents (2006 – 2015)

Type of document	Frequency	% *N* = 2260
Article	1612	71.33
Review	216	9.56
Letter	215	9.51
Editorial	85	3.76
Note	65	2.88
Article in Press	27	1.19
Short Survey	22	0.97
Conference Paper	18	0.80

### Country analysis

Authors from 124 different countries contributed to retrieved documents. The United States of America (USA) ranked first in the number of publications followed by India, South Africa, and the United Kingdom (UK). Table 2 shows the list of all countries that contributed to publications. There was a total of 243 (10.7%) articles with undefined affiliation. These articles include one or more authors with missing country affiliation or country affiliation of certain authors is not being updated by Scopus system at the date of data analysis. Table 3 shows the list of top 10 productive countries. The top 10 list included five European countries plus the USA, India, China, South Korea, and South Africa. Netherlands ranked 11^th^ and was outside the top 10 active list.

**Table 2:** List of all countries contributing to retreived data (2006 – 2015)

Country	Frequency	% *N* = 2260	Country	Frequency	% *N* = 2260	Country	Frequency	% *N* = 2260	Country	Frequency	% *N* = 2260
USA	517	22.88	Argentina	29	1.28	Zambia	7	0.31	Azerbaijan	2	0.09
India	269	11.90	Belarus	19	0.84	Austria	6	0.27	Central African Republic	2	0.09
South Africa	229	10.13	Denmark	19	0.84	Burkina Faso	6	0.27	Cuba	2	0.09
UK	186	8.23	Viet Nam	18	0.80	Cote d’Ivoire	6	0.27	Ecuador	2	0.09
China	158	6.99	Georgia	15	0.66	Lesotho	6	0.27	Jamaica	2	0.09
France	122	5.40	Poland	15	0.66	Botswana	5	0.22	Mozambique	2	0.09
Switzerland	117	5.18	Colombia	14	0.62	Bulgaria	5	0.22	Palestine	2	0.09
South Korea	102	4.51	Czech Republic	13	0.58	Cambodia	5	0.22	Sudan	2	0.09
Italy	100	4.42	Ethiopia	13	0.58	Chile	5	0.22	Swaziland	2	0.09
Germany	91	4.03	Romania	13	0.58	Egypt	5	0.22	Tunisia	2	0.09
Netherlands	85	3.76	Bangladesh	12	0.53	Guadeloupe	5	0.22	Venezuela	2	0.09
Spain	73	3.23	Singapore	12	0.53	Haiti	5	0.22	Albania	1	0.04
Peru	69	3.05	Malaysia	11	0.49	Lithuania	5	0.22	Croatia	1	0.04
Iran	64	2.83	Gambia	10	0.44	Mongolia	5	0.22	Djibouti	1	0.04
Belgium	62	2.74	Hungary	10	0.44	Congo	4	0.18	Dominican Republic	1	0.04
Japan	61	2.70	Namibia	10	0.44	Iceland	4	0.18	Micronesia	1	0.04
Sweden	56	2.48	Uganda	10	0.44	Ireland	4	0.18	French Guiana	1	0.04
Brazil	51	2.26	Ukraine	10	0.44	Kazakhstan	4	0.18	Ghana	1	0.04
Russian Federation	51	2.26	Kuwait	9	0.40	New Zealand	4	0.18	Guinea-Bissau	1	0.04
Portugal	48	2.12	Moldova	9	0.40	Algeria	3	0.13	Jordan	1	0.04
Australia	44	1.95	Nigeria	9	0.40	Armenia	3	0.13	Laos	1	0.04
Pakistan	43	1.90	Saudi Arabia	9	0.40	Benin	3	0.13	Lebanon	1	0.04
Taiwan	42	1.86	Greece	8	0.35	Cameroon	3	0.13	Oman	1	0.04
Thailand	40	1.77	Nepal	8	0.35	Finland	3	0.13	Puerto Rico	1	0.04
Mexico	36	1.59	Norway	8	0.35	Gabon	3	0.13	Rwanda	1	0.04
Canada	35	1.55	Tanzania	8	0.35	Honduras	3	0.13	Saint Kitts and Nevis	1	0.04
Estonia	32	1.42	Indonesia	7	0.31	Iraq	3	0.13	Slovenia	1	0.04
Latvia	32	1.42	Israel	7	0.31	Madagascar	3	0.13	Somalia	1	0.04
Turkey	32	1.42	Kenya	7	0.31	Malawi	3	0.13	Togo	1	0.04
Philippines	31	1.37	Luxembourg	7	0.31	Morocco	3	0.13	UAE	1	0.04
Hong Kong	30	1.33	Uzbekistan	7	0.31	Panama	3	0.13	Undefined	243	10.75

**Table 3:** Top ten productive countries along with their international collaboration and citation analysis (2006–2015)

SCR	Country	Frequency	% *N* = 2260	TC	C/A	*h*-index	NCC	SCP	%	TC	C/A	*h*-index	MCP	%	TC	C/A	*h*-index
1^st^	USA	517	22.88	15,483	29.95	61	75	147	28.43	3153	21.45	30	370	71.57	12,330	33.32	52
2^nd^	India	269	11.90	3341	12.42	29	39	216	80.30	1805	8.36	21	53	19.70	1536	28.98	18
3^rd^	S. Africa	229	10.13	8380	36.59	40	50	45	19.65	555	12.33	12	184	80.35	7825	42.53	39
4^th^	UK	186	8.23	4608	24.77	33	69	38	20.43	336	8.84	11	148	79.57	4272	28.86	33
5^th^	China	158	6.99	1460	9.24	19	30	110	69.62	713	6.48	16	48	30.38	747	15.56	13
6^th^	France	122	5.40	3116	25.54	25	74	33	27.05	327	9.91	9	89	72.95	2789	31.34	24
7^th^	Switzerland	117	5.18	5766	49.28	37	63	22	18.80	1015	46.14	10	95	81.20	4751	50.01	33
8^th^	S. Korea	102	4.51	3004	29.45	25	37	73	71.57	1161	15.90	19	29	28.43	1843	63.55	15
9^th^	Italy	100	4.42	3515	35.15	32	52	25	25.00	199	7.96	9	75	75.00	3316	44.21	31
10^th^	Germany	91	4.03	2837	31.18	28	55	21	23.08	132	6.29	5	70	76.92	2705	38.64	22

*SCR* standard competition rank

*TC* total citations

*C/A* number of citations per article. It is calculated by dividing the total number of citations by total number of publications retrieved for the assigned country

*NCC* number of collaborating countries

*SCP* single country publications (intra country collaboration)

*MCP* multiple country publications (inter country collaboration)

*h*-*index* Hirsh index

*USA* United States of America

*UK* United Kingdom

*S. Africa* South Africa

*S. Korea* South Korea

When top productive countries were stratified by number of citations per article, Switzerland ranked first followed by South Africa and Italy. Publications from India and China had the lowest number of citations per article. Articles produced by international collaboration (MCP) had higher number of citations per article compared with articles produced without international collaboration (SCP). This indicates that international collaboration is important in increasing number of citations. Interestingly, countries with the lowest percentage of international collaboration, such as China and India, had the lowest number of citations per article. When data for top ten countries were stratified by population size, Switzerland ranked first followed by South Africa and the UK (Table 4). When data were stratified based on GDP, South Africa ranked first followed by Switzerland and India.

**Table 4:** Productivity of top ten active countries stratified by population size and GDP

SCR	Country	Frequency	% *N* = 2260	Number of population in millions	Number of articles per 100 million population^a^(R)	GDP in trillions	Number of articles per one trillion GDP^b^ (R)
1^st^	USA	517	22.88	321.4	160.86 (7)	17.95	28.80 (9)
2^nd^	India	269	11.90	1311	20.52 (9)	2.074	131.41 (3)
3^rd^	S. Africa	229	10.13	54.96	416.67 (2)	0.3128	731.63 (1)
4^th^	UK	186	8.23	65.14	285.54 (3)	2.849	65.29 (4)
5^th^	China	158	6.99	1371	11.52 (10)	10.87	14.54 (10)
6^th^	France	122	5.40	66.81	182.61 (5)	2.422	50.37 (7)
7^th^	Switzerland	117	5.18	8.287	1411.85 (1)	0.6647	175.94 (2)
8^th^	S. Korea	102	4.51	50.81	200.79 (4)	1.929	55.19 (5)
9^th^	Italy	100	4.42	60.80	164.47 (6)	1.815	55.10 (6)
10^th^	Germany	91	4.03	81.41	111.78 (8)	3.356	27.12 (8)

*SCR* Standard competition rank

*USA* United States of America

*UK* United Kingdom

*S. Africa* South Africa

*S. Korea* South Korea

*R* rank

*GDP* Gross Domestic Product

*MDR, XDR, and TDR-TB* multi-, extensively, and totally drug resistant tuberculosis

^a^calculated by dividing number of publications for a particular country by its population size then multiplying by 100

^b^calculated by dividing the number of publications of a particular country by its GDP in trillions

**Fig. 4:**
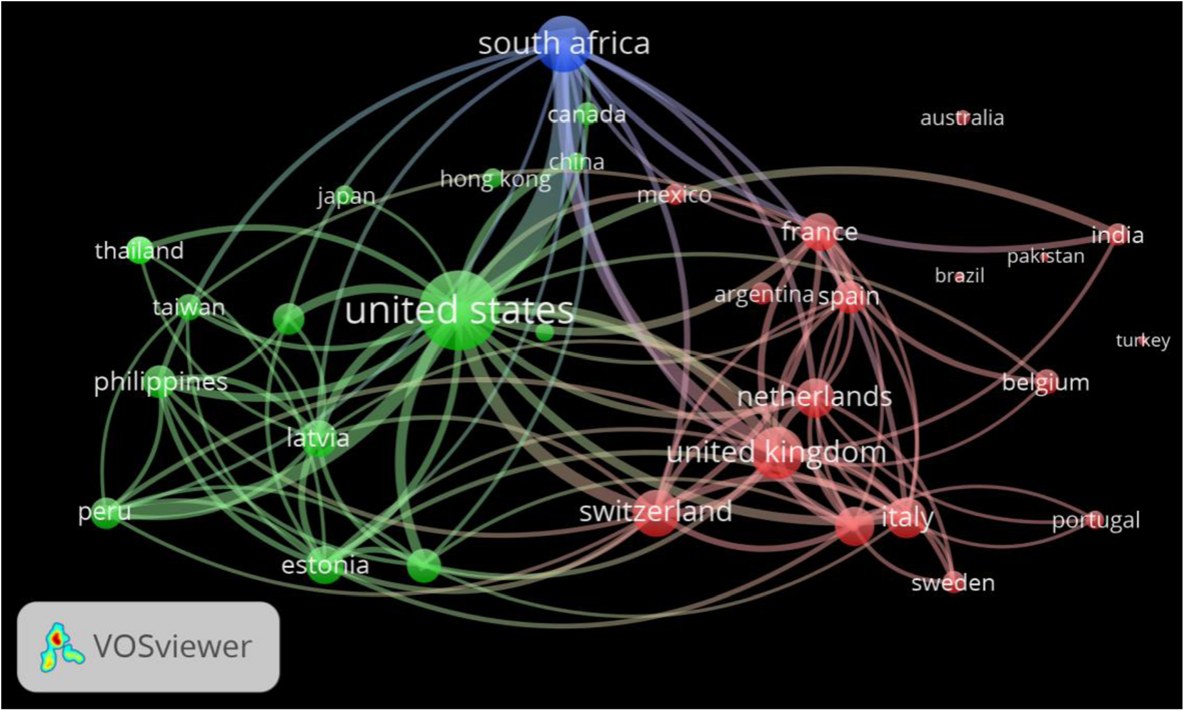
Network visualization map of country co-authorships. Legend: Countries with a minimum of 20 published articles were included. The map includes 32 countries in three clusters. Number of co-authorships was highest for the USA followed by South Africa and the UK. Strong collaborations were found between the following pairs of countries: *USA – South Africa* (link strength = 109), *USA – Peru* (link strength = 47), *South Africa – UK* (link strength = 42), *USA – Switzerland* (link strength = 42), *USA – UK* (link strength = 36), *USA – Russian Federation* (link strength = 30). *USA – South Korea* (link strength = 26), *USA – China* (link strength = 24), *USA – Latvia* (link strength = 25), and *UK – Germany* (link strength = 23)

Visualization of international collaboration among countries with a minimum productivity of twenty documents is shown in Fig. 4. Visualization map shows the extent and strength of international collaboration between any two countries. The extent of international collaboration for any country is assessed by the size of circle assigned for that country while the strength of collaboration between any two countries is assessed by the thickness of the line connecting the two countries. Strong collaborations were found between the following pairs of countries: *USA – South Africa* (link strength = 109), *USA – Peru* (link strength = 47), *South Africa – UK* (link strength = 42), *USA – Switzerland* (link strength = 42), *USA – UK* (link strength = 36), *USA – Russian Federation* (link strength = 30). *USA – South Korea* (link strength = 26), *USA – China* (link strength = 24), *USA – Latvia* (link strength = 25), and *UK – Germany* (link strength = 23).

### Productivity from HBC MDR-TB

**Fig. 5:**
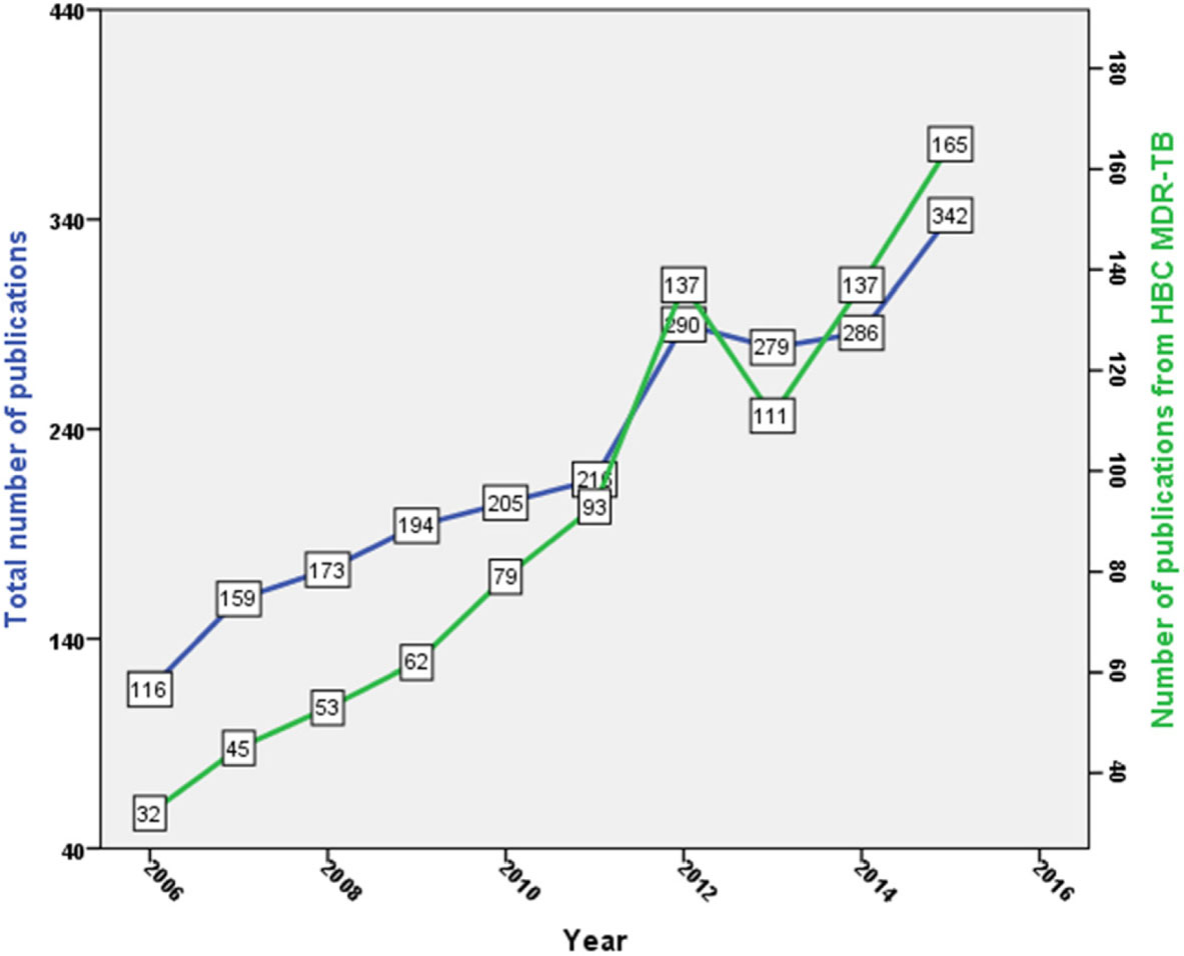
Growth of publications from HBC MDR-TB (green line) compared with worldwide productivity (2006–2015). Key: Green line: HBC MDR-TB productivity. Blue line: Worldwide productivity

Three of HBC (India, China, and South Africa) were in the top ten list. On the other hand, seven HBC (N. Korea, Myanmar, Angola, Kyrgyzstan, Papua New Guinea, Tajikistan, and Zimbabwe) had no contribution at all. The remaining HBC (20 countries) were shown in Table 5. The majority of contributing HBC MDR-TB had more than 40% of their publications produced through international collaboration. The exception was Indonesia. The total number of publications produced by HBC MDR-TB, including China, India, and South Africa, was 914 articles. The growth of publications from HBC MDR-TB was almost parallel to worldwide productivity (Fig. 5). However, the gap between the two curves get narrower with time and the growth rate of publications from HBC MDR-TB exceeded that of the worldwide productivity in the last few years. This is due to research productivity from China, India, and South Africa which are considered within the HBC MDR-TB. In 2006, productivity from HBC MDR-TB was almost one forth that of worldwide while in 2015 the productivity of HBC MDR-TB became half that of worldwide. Poisson regression model showed that productivity from HBC MDR-TB was a significant predictor (*p* < 0.001) of worldwide productivity. The model indicated that for each article produced from HBC MDR-TB, the worldwide productivity increased by 1.01.

**Table 5:** Productivity of HBC MDR-TB along with their citation analysis and international collaboration

Country^a^	Frequency	(%) *N* = 2260	TC	C/A	*h*-index	NCC	SCP	% of SCP	MCP	% of MCP
		Top HBC MDR-TB countries by estimated absolute number (in alphabetical order)								
Bangladesh	12	0.53	294	24.50	6	7	5	41.67	7	58.33
DR Congo	4	0.18	9	2.25	2	5	0	0.00	4	100.00
Ethiopia	13	0.58	172	13.23	6	6	7	53.85	6	46.15
Indonesia	7	0.31	14	2.00	2	5	5	71.43	2	28.57
Kazakhstan	4	0.18	9	2.25	1	1	0	0.00	4	100.00
Kenya	7	0.31	309	44.14	4	14	2	28.57	5	71.43
Mozambique	2	0.09	295	147.50	2	5	0	0.00	2	100.00
Nigeria	9	0.40	32	3.56	3	9	4	44.44	5	55.56
Pakistan	43	1.90	283	6.58	11	29	25	58.14	18	41.86
Philippines	31	1.37	1705	55.00	14	33	5	16.13	26	83.87
Russian F.	51	2.26	1983	38.88	23	36	8	15.69	43	84.31
Thailand	40	1.77	815	20.38	12	29	18	45.00	22	55.00
Ukraine	10	0.44	61	6.10	6	18	3	30.00	7	70.00
Uzbekistan	7	0.31	189	27.00	5	11	0	0.00	7	100.00
Viet Nam	18	0.80	514	28.56	8	28	3	16.67	15	83.33
		Countries with the highest number of TB case rates *per capita*								
Azerbaijan	2	0.09	443	221.50	1	9	0	0.00	2	100.00
Belarus	19	0.84	469	24.68	9	33	0	0.00	19	100.00
Peru	69	3.05	2501	36.25	21	28	9	13.04	60	86.96
Moldova	9	0.40	108	12.00	7	22	0	0.00	9	100.00
Somalia	1	0.04	8	8.00	1	3	0	0.00	1	100.00

*HBC MDR-TB* High Burden Countries with Multidrug Resistant Tuberculosis

*TC* total citations

*C/A* average number of citations per article. It is calculated by dividing the total number of citations by total number of publications retrieved for the assigned country

*NCC* number of collaborating countries

*SCP* single country publications (intra country collaboration)

*MCP* multiple country publications (inter country collaboration)

*h*-*index* Hirsh index

^a^Countries were listed alphabetically. Countries that were listed in top ten list were not listed in this table: China, India, and South Africa. The following countries have zero contribution: N. Korea, Myanmar, Angola, Kyrgyzstan, Papua New Guinea, Tajikistan, Zimbabwe

### Institutions/ Organizations

Top ten productive institutions/ organizations are shown in Table 6. The WHO ranked first followed by Centers for Disease Prevention and Control (CDC) and Harvard Medical School. The total productivity from Harvard Medical School and Harvard School of Public Health was 98 articles which is greater than that of CDC and lower than that of WHO. The top list included five American institutions/ organizations, three South African, two Italian, and one international organization (WHO). The impact of publications from top ten productive institutions/ organizations was assessed indirectly by the percentage of highly cited articles for each institutions/ organization. *Albert Einstein College of Medicine of Yeshiva University* (USA) ranked first in the percentage of highly cited articles followed equally by the two Italian institutions, *Università degli Studi di Sassari,* and *IRCCS Fondazione Salvatore Maugeri.*

**Table 6:** Top ten active institutions/ organizations in MDR, XDR, and TDR-TB publications (2006–2015)

SCR^a^	Institution/ Organization	Country	Frequency	% *N* = 2260	HCA	% of HCA^b^
1^st^	*Organisation Mondiale de la Sante*	WHO	104	4.60	35	33.65
2^nd^	*Centers for Disease Control and Prevention*	CDC (USA)	89	3.94	31	34.83
3^rd^	*Brigham and Women’s Hospital*	USA	74	3.27	18	24.32
4^th^	*Harvard Medical School*	USA	73	3.23	20	27.40
5^th^	*Universiteit Stellenbosch*	S. Africa	61	2.70	23	37.70
6^th^	*University of Cape Town*	S. Africa	54	2.39	16	29.63
7^th^	*Albert Einstein College of Medicine of Yeshiva University*	USA	44	1.95	25	56.82
8^th^	*South African Medical Research Council*	S. Africa	43	1.90	17	39.53
9^th^	*Universita degli Studi di Sassari*	Italy	42	1.86	18	42.86
9^th^	*IRCCS Fondazione Salvatore Maugeri*	Italy	42	1.86	18	42.86
9^th^	*Partners in Health*	USA	42	1.86	12	28.57

^a^SCR: standard competition rank. Equal countries were given the same ranking number, and then a gap is left in the ranking numbers

^b^HCA: number of highly cited articles. Articles with ≥ 30 citations were considered HCA. Percentage of HCA was calculated by dividing total number of HCA by total number of publications for each institution/ organization assigned

### Journals

Journals publishing at least 20 articles on MDR, XDR, TDR-TB are shown in Table 7. The total number of publications produced by journals in the list was 975 (43.14%) with a total IF of 7494.27 which gives an average of 7.68 per article. The *International Journal of Tuberculosis and Lung Diseases* ranked first followed by *PloS One*. Most journals in the list have IF. *PloS One* journal was the only multidisciplinary journal present in the list. The other ones in the list were within the scope of infection and general medicine. *Lancet* and *New England Journal of Medicine* (NEJM) had the highest IF and both are in general medicine. The top list included one Chinese and one Japanese journal while the remaining journals are Europeans or American ones.

**Table 7:** Journals publishing at least 20 articles on MDR, XDR, and TDR – TB (2006–2015)

Journal	Country	Frequency	%	IF	TIF
*International Journal of Tuberculosis and Lung Disease*	France	212	9.38	2.315	490.78
*Plos One*	USA	123	5.44	3.54	435.42
*European Respiratory Journal*	Switzerland	81	3.58	8.332	674.892
*Emerging Infectious Diseases*	USA	61	2.70	6.99	426.39
*Journal of Clinical Microbiology*	USA	54	2.39	3.993	215.622
*Clinical Infectious Diseases*	USA	50	2.21	8.736	436.8
*Antimicrobial Agents and Chemotherapy*	USA	41	1.81	3.34	136.94
*Lancet*	UK	41	1.81	44.002	1804.082
*Journal of Antimicrobial Chemotherapy*	UK	38	1.68	4.919	186.922
*BMC Infectious Diseases*	UK	37	1.64	2.690	99.53
*International Journal of Mycobacteriology*	USA	37	1.64	N/A	0
*Indian Journal of Tuberculosis*	India	30	1.33	N/A	0
*Lancet Infectious Diseases*	UK	27	1.19	21.371	577.017
*American Journal of Respiratory and Critical Care Medicine*	USA	26	1.15	13.118	341.068
*International Journal of Antimicrobial Agents*	Netherlands	26	1.15	4.079	106.054
*New England Journal of Medicine*	USA	25	1.11	59.558	1488.95
*Tuberculosis*	USA	25	1.11	2.952	73.8
*Kekkaku*	Japan	21	0.93	N/A	0
*Chinese Journal of Tuberculosis and Respiratory Diseases*	China	20	0.88	N/A	0
		975 (43.14%)			7494.267

*IF* impact factor

*TIF* total impact factor obtained by multiplying the impact factor of the journal by the number of articles published by that journal = 7494.267

**Fig. 6:**
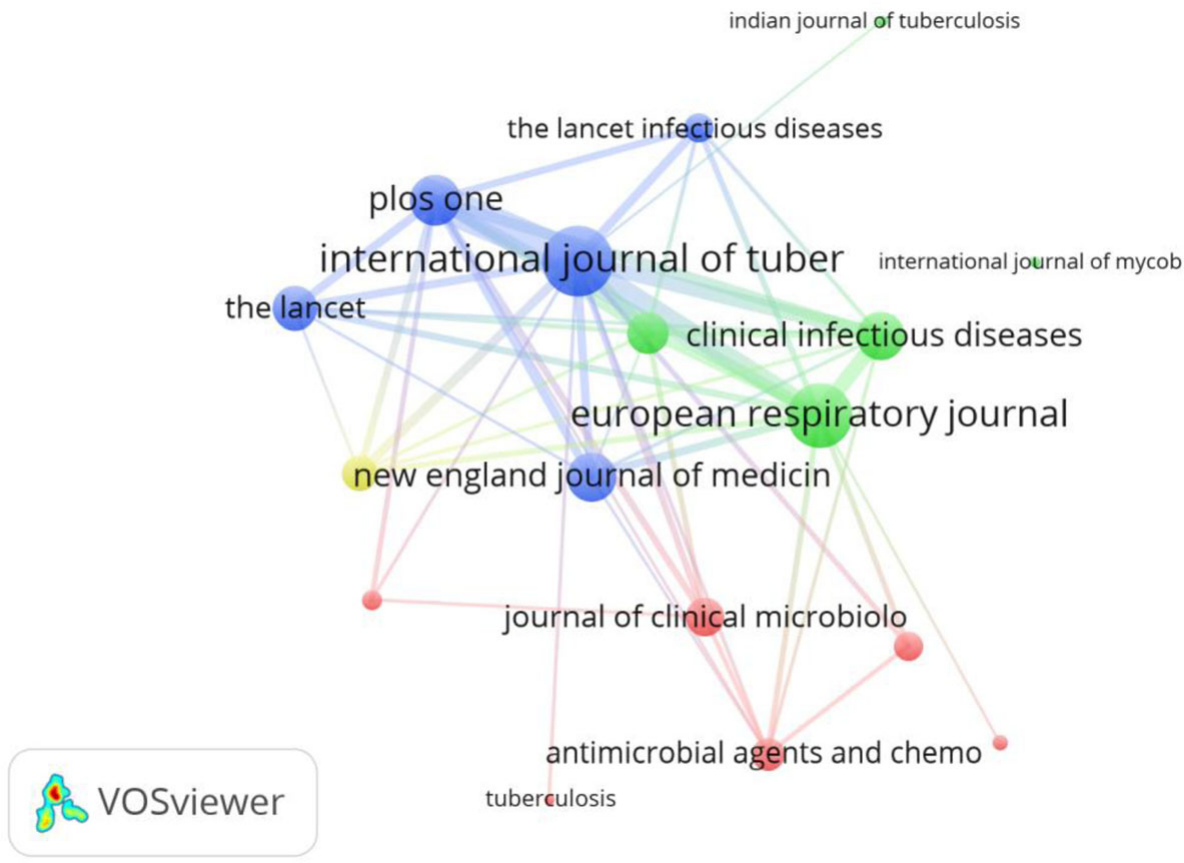
Network visualization map of journal citations. Legend: Journals with a minimum of 20 articles published on MDR, XDR, TDR-TB were included. *International Journal of Tuberculosis and Lung Disease* had the highest number of citations followed by *European Respiratory Journal* and *PloS One*. The number of citations is correlated with the circle size assigned for the journal

**Fig. 7:**
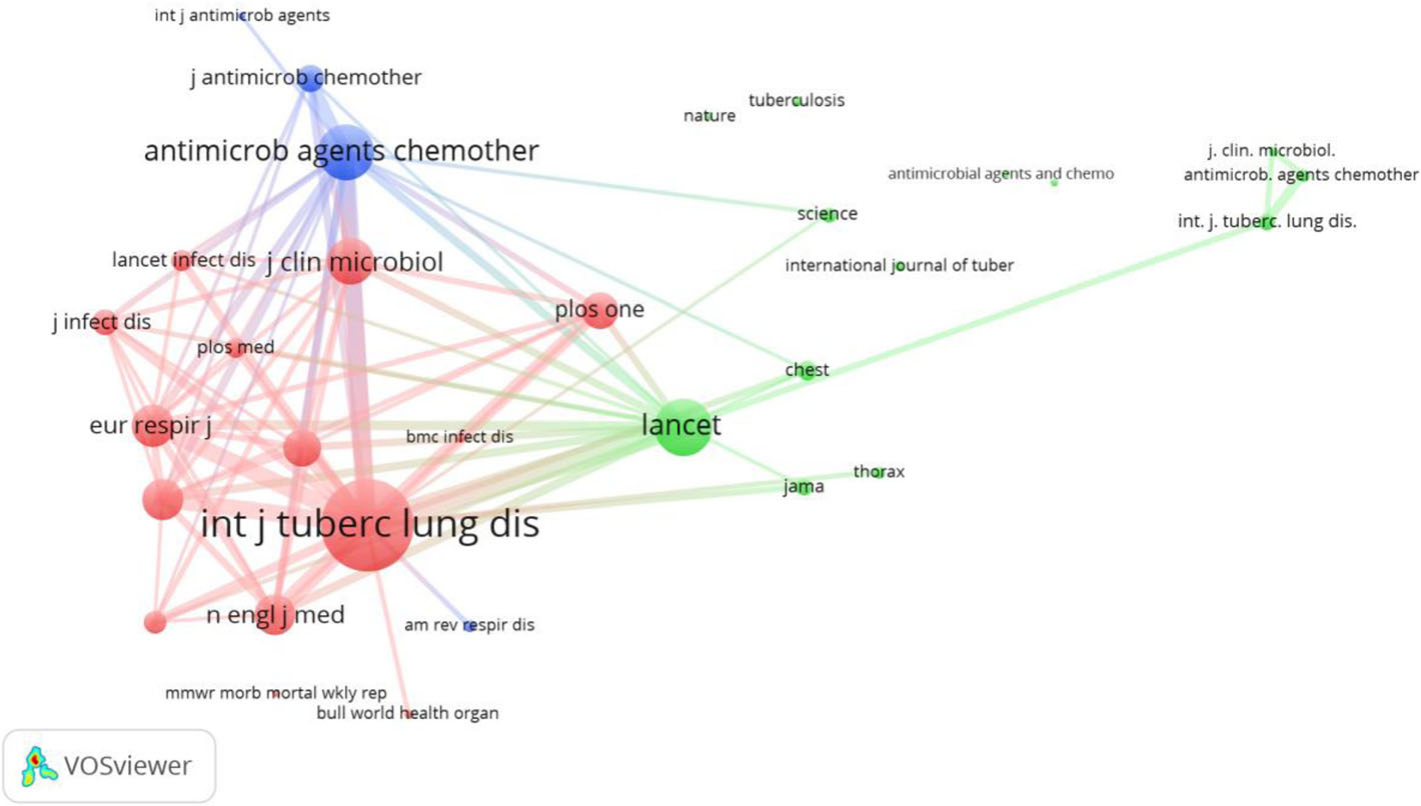
Journal co-citation analysis. Legend: Journals with a minimum of 200 citations were included. Twenty journals are shown in the network visualization map to make the map readable and less crowded. *International Journal of Tuberculosis and Lung Disease* is commonly co-cited with *Lancet* (link strength = 6842), *Journal of Clinical Microbiology* (link strength = 5767), *Antimicrobial Agents and Chemotherapy* (link strength = 5052), *NEJM* (link strength = 5161), *Clinical Infectious Disease* (link strength = 4882), and *American Journal of Respiratory and Critical Care Medicine* (link strength = 4515)

Citation analysis of most productive journals is depicted as a network visualization map (Fig. 6). *International Journal of Tuberculosis and Lung Disease* had the highest number of citations followed by *PloS One* and *European Respiratory Journal*. The number of citations is correlated with the circle size assigned for the journal. Co-citation analysis for most productive journals is shown in Fig. 7. Journals with a minimum of 200 citations were included. *International Journal of Tuberculosis and Lung Disease* is commonly co-cited with *Lancet* (link strength = 6842), *Journal of Clinical Microbiology* (link strength = 5767), *Antimicrobial Agents and Chemotherapy* (link strength = 5052), *NEJM* (link strength = 5161), *Clinical Infectious Disease* (link strength = 4882), and *American Journal of Respiratory and Critical Care Medicine* (link strength = 4515).

### Authors

**Fig. 8:**
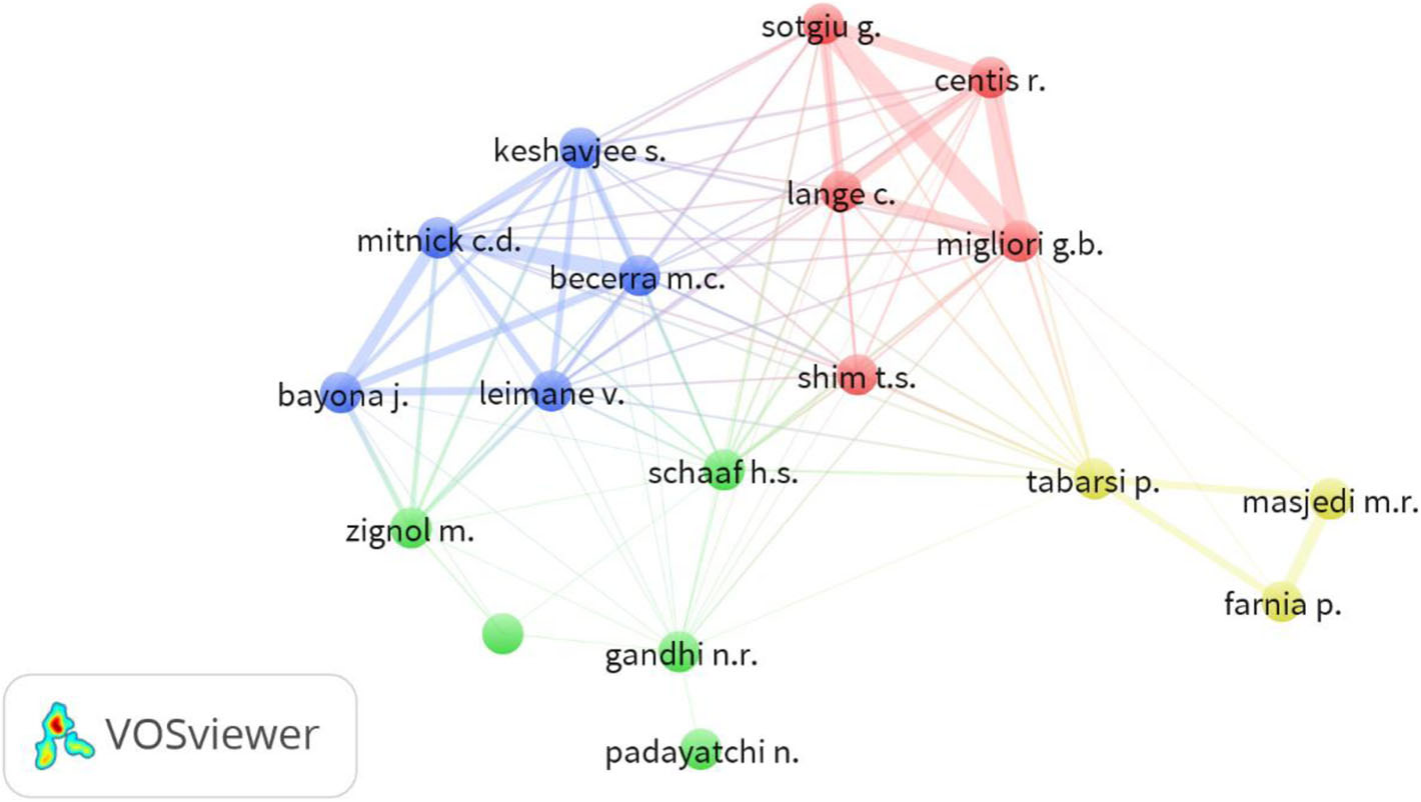
Network visualization for author co-authorships (collaboration). Legend: Authors with a minimum productivity of 20 articles were included. Examples of strong collaboration were seen among the following authors: *Migliori, G.B. - Sotgiu, G*. (link strength = 37), *Migliori, G.B. - Centis, R.* (link strength = 37), *Mitnick, C.D. - Becerra, M.C.* (link strength = 22), *Migliori, G.B. - Lange, C.* (link strength = 22), *Sotgiu, G. - Centis, R.* (link strength = 25), *Mitnick, C.D. - Bayona, J.* (link strength = 16), and *Masjedi, M.R. - Farnia, P.* (link strength = 17). The strength of collaboration is relative to the thickness of connection between any two researchers. Authors with similar circle color are considered a cluster, i.e. have close collaboration

A total of 8246 authors contributed to the publications, giving an average of 3.65 authors per article. Authors who had published at least 20 articles are shown in Table 8. The list included five authors from the USA, three authors from Italy, three authors from Iran, two authors from South Africa, two from WHO, one from China, one from Latvia, one from Germany, one from Switzerland, one from Sweden, and one with two affiliations, USA/ UAE. Author collaboration among top active authors is depicted in Fig. 8. Examples of strong collaboration were seen among the following authors: *Migliori, G.B. - Sotgiu, G*. (link strength = 37), *Migliori, G.B. - Centis, R.* (link strength = 37), *Mitnick, C.D. - Becerra, M.C.* (link strength = 22), *Migliori, G.B. - Lange, C.* (link strength = 22), *Sotgiu, G. - Centis, R.* (link strength = 25), *Mitnick, C.D. - Bayona, J.* (link strength = 16), and *Masjedi, M.R. - Farnia, P.* (link strength = 17). The strength of collaboration is relative to the thickness of connection between any two researchers. Authors with similar circle color are considered a cluster, i.e. have close collaboration.

**Table 8:** Top active authors with a minimum contribution of 20 articles (2006 – 2015)

Author	Frequency	% *N* = 2260	Affiliation as appeared in Scopus profile
Migliori, G.B.	63	2.79	*IRCCS Fondazione Salvatore Maugeri, Pavia, Italy*
Mitnick, C.D.	36	1.59	*Partners in Health, Boston, United States*
Sotgiu, G.	42	1.86	*Universita degli Studi di Sassari, Department of Biomedical Sciences, Sassari, Italy*
Becerra, M.C.	29	1.28	*Harvard Medical School, Department of Global Health and Social Medicine, Boston, United States*
Keshavjee, S.	31	1.37	*Harvard Medical School Center for Global Health Delivery, Dubai, United Arab Emirates*
Centis, R.	39	1.73	*IRCCS Fondazione Salvatore Maugeri, Pavia, Italy*
Bayona, J.	25	1.11	*Harvard Medical School, Department of Global Health and Social Medicine, Boston, United States*
Farnia, P.	25	1.11	*Shahid Beheshti University of Medical Sciences, Tehran, Iran*
Gandhi, N.R.	27	1.19	*Rollins School of Public Health, Atlanta, United States*
Leimane, V.	26	1.15	*Riga East University Hospital, Riga, Latvia*
Masjedi, M.R.	25	1.11	*Shahid Beheshti University of Medical Sciences, Chronic Respiratory Disease Research Center, Tehran, Iran*
Yew, W.W.	26	1.15	*Chinese University of Hong Kong, Stanley Ho Centre for Emerging Infectious Diseases, Hong Kong, China*
Zignol, M.	24	1.06	*Organisation Mondiale de la Sante, Global TB Programme, Geneve, Switzerland*
Lange, C.	27	1.19	*Forschungszentrum Borstel - Zentrum fur Medizin und Biowissenschaften, Borstel, Germany*
Padayatchi, N.	25	1.11	*Centre for the AIDS Programme of Research in South Africa, Congella, South Africa*
Tabarsi, P.	23	1.02	*Shahid Beheshti University of Medical Sciences, Tehran, Iran*
Victor, T.C.	20	0.88	*Universiteit Stellenbosch, Division of Molecular Biology and Human Genetics, Stellenbosch, South Africa*
Koh, W.J.	22	0.97	*SungKyunKwan University, School of Medicine, Department of Medicine, Suwon, South Korea*
D’Ambrosio, L.	20	0.88	*Public Health Consulting Group, Lugano, Switzerland*
Falzon, D.	20	0.88	*Organisation Mondiale de la Sante, Global TB Programme, Geneve, Switzerland*
Hoffner, S.	20	0.88	*Karolinska University Hospital, Department of Microbiology, Tumor and Cell Biology, Stockholm, Sweden*
Shah, N.S.	20	0.88	*Albert Einstein College of Medicine of Yeshiva University, Department of Medicine, New York, United States*

### Highly cited articles

Table 9 shows the top 20 highly cites articles. The highest number of citations obtained was 1069. The article titled *“Extensively drug-resistant tuberculosis as a cause of death in patients co-infected with tuberculosis and HIV in a rural area of South Africa”* received the highest number of citations. Four articles from the top 20 cited articles were published in *NEJM*, three articles were published in *Lancet*, and two were published in *Lancet Infectious Diseases*. The topics covered in highly cited articles range from general public health impact of MDR, XDR, TDR-TB to results of newly approved drugs for treatment of MDR-TB. The top 20 cited articles included five review articles, one conference paper, one letter, one short survey, and the remaining were research articles.

**Table 9:** Top highly cited articles on MDR, XDR, TDR-TB (2006–2015)

Reference	Number of citations	Document Type	Reference	Number of citations	Document Type
[53]	1069	Article	[54]	264	Article
[55]	587	Review	[56]	262	Article
[57]	443	Article	[58]	261	Article
[59]	435	Review	[60]	257	Short Survey
[61]	424	Article	[62]	253	Letter
[63]	374	Article	[64]	246	Review
[65]	340	Article	[66]	236	Article
[67]	297	Review	[68]	226	Article
[7]	290	Article	[69]	219	Article
[70]	284	Conference Paper	[71]	214	Review

## Discussion

In this study, a bibliometric overview of publications on MDR, XDR, and TDR-TB was presented. Our study showed a steady increase on worldwide publications on MDR, XDR and TDR-TB suggesting that this problem is a global health problem and can be a public health burden if the current situation continues as is [34, 35]. The value of *h*-index of retrieved articles in the past decade supports the fact that this problem is spreading and is being cited heavily in the literature. Analysis of Scopus database for articles with keywords pertaining to infectious diseases showed six fold increase in the number of publications from 2006 to 2015. Therefore, it is possible that part of the increase in MDR, XDR, and TDR-TB publications is due to the overall growth of publications with time in the field of infections and infectious diseases. However, analysis of Scopus database for publications in the past decade showed that the number of publications on TB was less than doubled in the past decade while the number of publications on TB resistance had increased by more than three folds in the past decade suggesting that resistance is increasing at a rate higher than that of TB in general.

According to Global Tuberculosis Report 2015, a total of approximately 10 million incident cases of TB were recorded in 2014, mostly in Asia and Africa [3, 36]. This explains the presence of China, India, South Korea and South Africa in the top ten productive countries. A study in China found that 25% of surveyed TB patients had TB that was resistant to isoniazid, rifampin, or both, and 1 out of 10 had MDR tuberculosis [37]. Reports from India about MDR, XDR and TDR –TB have been published that raises calls of immediate response by Indian medical community [38]. South Africa has also reported many cases of MDR, XDR and TDR-TB [39, 40]. The Global Tuberculosis Report further explains that 11–13% of new incident tuberculosis cases in 2014 were among HIV positive people. This might explain the presence of two articles in the most cited list that discuss tuberculosis and MDR in HIV patients. Actually, a study has reported that tuberculosis, drug abuse, HIV, and MDR-TB are devastating epidemics in some countries in Asia and urgent action is needed [41]. Our study showed that three countries of HBC MDR-TB ranked among the top ten active countries. In general, the publication from HBC MDR-TB was parallel to that of worldwide productivity suggesting that there is a real interest and concern regarding this subject. Out of the twenty HBC MDR-TB, Russian Federation and Peru had noticeable contribution. Russian Federation is considered by the WHO as one of 30 countries described as HBC TB [42].

International collaboration was common among most countries in the top productive countries. Despite that initiation of successful collaboration between Western rich countries and some poor countries with high TB prevalence has been reported [43], countries like China, India and South Korea where TB and TB-resistant cases are found showed lower international collaboration in TB drug resistance research. It could be the language barrier particularly for Chinese and Korean scientists. Other possible explanations include lack of collaboration between these countries and European and American countries in most other medical research fields. Collaboration among authors in different countries is important in advancing science and in combating TB through exchange of expertise and knowledge. A study indicated that public – private collaboration can produce successful detection and therapy of TB cases [44]. Another study indicated that governmental – NGOs collaboration in poor countries like Bangladesh is an effective strategy in detection and eradication of TB [45]. Antimicrobial resistance is a true challenge for international efforts to stop and reverse TB worldwide [46]. Preparing for this challenge requires collaboration, use of advanced molecular biology techniques, development of new pharmaceuticals, and worldwide research activity on TB.

Exploration of the top ten cited articles on MDR, EDR and TDR-TB reveals much to be planned for in this regard. It is true that TB is a major single killer among all infectious diseases, but co-infection of TB with HIV makes the prognosis even worse which requires the availability of potent anti-TB drugs and combating development of resistance to these drugs to promote better prognosis for patients co-infected with HIV and TB. Some authors described the presence of HIV and MDR-TB as a perfect storm with disastrous consequences [47]. Effective diagnostic tools for screening and early detection of TB and MDR-TB is an important strategy in combating drug resistant TB [48, 49]. The development of such cost-effective diagnostic techniques is urgent given that the majority of TB patients have no access to drug-resistance screening to optimize their therapy [49]. No wonder that an article about the feasibility and efficacy of a diagnostic techniques was among top ten cited articles [48]. One more important of concern in drug – resistant TB global public health challenge is the search for new drugs to overcome reported MDR-TB. One such drug is diarylquinoline TMC207 which is still under clinical investigation for MDR-TB cases [50].

No doubt that drug resistance TB deserves a lot of research and the scientific community needs to be aware where worldwide research on this topic is standing. Our study is being strong in the fact that it is the first to give a bibliometric overview on MDR, XDR and TDR – TB publications. However, missing some articles is a possibility due to unindexed journal publication or accuracy of search strategy despite that the authors have done their best to avoid false negative and false positive results as described in the methodology. Furthermore, the use of title search strategy instead of title/abstract strategy created some pros and cons. Title search strategy created minimum false positive results, but might create some false negative results that need to be taken into consideration. No bibliometric study is 100% accurate and perfect and it is always a snapshot of the current situation based on certain keywords used in data collection. In addition, no electronic database is considered perfect for data collection. System updates, different affiliations, different name spelling, and sometimes missing data can be found in electronic databases commonly used in bibliometric studies. Finally, it should be emphasized that definitions pertaining to resistance terminology are not standardized. However, the definitions presented in this study are the most common ones. Limitations in this study are similar in nature to those present and listed in most bibliometric studies [21–28]. Despite all these limitations, this study was meant to fill gap in literature and knowledge on MDR, XDR, and TDR –TB in general hoping that this study will be of value to those in the field. Furthermore, this is the first bibliometric study on MDR, XDR, and TDR-TB despite that several bibliometric studies on TB were carried out [51, 52].

## Conclusions

Our study showed that publications on MDR, XDR and TDR – TB have increased in the past decade. The majority of articles on MDR, XDR and TDR-TB were published in journals with high IF, suggestive of importance of the topic to clinicians and researchers. Search for feasible, accurate, cost effective diagnostic tests, and new anti TB drugs are hot topics in this field. International collaboration in this field was evident for most countries. The exchange of expertise, ideas and technology is of paramount importance in this field especially that TB is common in poor countries that lack these expertise.

## Abbreviations

WHO: World Health Organization
CDC: Centers for Disease Prevention and Control
TB: Tuberculosis
MDR: Multidrug resistant
XDR: Extensively resistant or extremely resistant
TDR: Totally resistant
HBC: High burden countries
HBC-TB: High burden courtiers – tuberculosis
HBC MDR-TB: High burden countries with multidrug resistant tuberculosis
